# The effects of a caffeine-containing beverage on neuromuscular performance during a multi-joint, lower body power exercise

**DOI:** 10.1186/1550-2783-9-S1-P22

**Published:** 2012-11-19

**Authors:** Meghan McCann, Amanda Wright, Stephen Siegle, Jenna Veldhuizen, Stephanie Wojton, Kelsey Jacobs, Eric Kuklinski, Tyler Krings, Elizabeth Scheckel, Carly Homan, Emily Kammerer, Dawn Anderson, Lonnie Lowery

**Affiliations:** 1 Department of Health, Exercise and Rehabilitative Sciences, Winona State University, Winona, MN 55987, USA

## Background

Current research has shown varied results when comparing the effects of caffeinated beverages on explosive exercise movements. We hypothesized that lower body muscular explosiveness would be significantly increased (p < 0.05) after Redline^®^ energy drink ingestion versus a similar placebo (PLB) drink in recreationally active subjects (n=16).

## Methods

After a day of dietary control and caffeine abstinence, otherwise-fasted participants performed four separate, strict squat jumps (SJ) under both conditions 48 – 96 hours apart. The variables measured included peak power (POW), peak force (FOR), peak velocity (VEL), maximal displacement (DSP), and maximal rate of force development (RFD) in the SJ for both Redline^®^ energy drink and PLB trials.

## Results

These preliminary data illustrated a significant increase in peak velocity in the Redline^®^ energy drink condition versus PLB (Redline® 2.35± 0.36 m/s vs. PLB 2.29± 0.34 m/s [p= 0.033]). All other variables were regarded as non-significant.

## Conclusion

Our early findings only partially support our hypothesis because all but one variable was unaffected during the squat jump. Future research should focus on potential differences between upper- and lower-body power exercises as they respond to caffeine-related interventions.

